# Acupuncture for Premenstrual Syndrome at Different Intervention Time: A Systemic Review and Meta-Analysis

**DOI:** 10.1155/2019/6246285

**Published:** 2019-06-25

**Authors:** Jiayuan Zhang, Liu Cao, Yunxia Wang, Yuxia Jin, Xiao Xiao, Qi Zhang

**Affiliations:** ^1^Chengdu University of Traditional Chinese Medicine, No. 37 Shierqiao Road, Jinniu District, Chengdu, Sichuan 610075, China; ^2^Sichuan Integrative Medicine Hospital, South Renmin Road, Wuhou District, Chengdu, Sichuan 610041, China

## Abstract

**Background:**

Premenstrual syndrome (PMS) is one of the most common gynecological conditions with no standard modern therapeutic schedule. Some studies have reported the effects of acupuncture in treating PMS, but the intervention time varies. This review evaluated the efficacy of acupuncture for patients with PMS and the appropriate time to initiate acupuncture therapy. The review has been registered on the “PROSPERO” website; the registration number is CRD42018109724.

**Methods:**

A comprehensive literature search was performed on 9 electronic databases from the time of inception to September 2018. RCTs studies on acupuncture for PMS compared with medication, sham acupuncture, or no treatment were included. Statistical analysis and investigation of heterogeneity source were carried out using RevMan5. 3.

**Results:**

A total of 15 studies, comprising of 1103 cases, were included. Overall, acupuncture significantly increased the effective rate of PMS compared with medicine and sham acupuncture. Subgroup analyses showed no significant difference among different intervention time to start acupuncture treatment. Among the acupoints involved in the treatment of PMS, SP6, LR3, and RN4 were the most commonly used.

**Conclusions:**

The current meta-analysis reveals that acupuncture leads to better effective rate, but the intervention time has no significant effect on the efficacy of acupuncture treatment for PMS. SP6, LR3, and RN4 are the most commonly used acupoints in treating PMS. However, large-scale, case-control studies with rigorous designs are required to provide more accurate evidence.

## 1. Introduction

Premenstrual syndrome (PMS) is characterized by repeated emotional, behavioral, and physical disorders during the luteal phase and is alleviated by the onset of menstruation [1]. Globally, 50%-80% of women experience premenstrual syndrome, and 30%-40% of them present with severe symptoms that affect physical as well as mental health which require treatment [2]. The etiology of PMS is unknown, and the pathogenesis is not fully understood. Presently, pharmacologic intervention for PMS includes hormone therapy and symptomatic treatment, using progesterone, oryzanol, vitamins, and oral antianxiety antidepressants [3]. Due to limited evidence on the efficacy of sustained progesterone [4] and the side effects of antidepressant and anxiolytics, in some cases, alternative therapies are recommended for patients with PMD. Acupuncture is one of the most commonly used therapies.

Numerous clinical randomized controlled trials and several meta-analyses [5, 6] have been performed to study the efficacy of acupuncture in the treatment of PMS, but there is no previous meta-analysis on the appropriate intervention time of acupuncture in treating PMS which is highly controversial and there is no uniform standard.

It is well known that the onset of premenstrual syndrome is periodic. Modern experiments have confirmed that time affects the efficacy of acupuncture [7–9]. Acupuncture modulates the “yin-yang balance” of the human body by stimulating the acupuncture points with fine needles inserted into the skin. Traditional Chinese medicine believes that the occurrence of PMS is related to the periodic yin-yang changes in the menstrual cycle. Therefore, some clinicians attach importance to intervention time when treating PMS with acupuncture according to the rules of yin-yang changes. However, the choice of intervention time is often based on traditional theory and experience which is lack of evidence-based medical evidence.

This meta-analysis compared the efficacy of acupuncture group and control group (including drugs, placebo acupuncture, or no treatment) in patients with PMS. It further compared the effectiveness of different intervention times in the acupuncture group to determine the best acupuncture treatment intervention time. The frequency of acupoints used to treat PMS in the included studies was also counted in this meta-analysis.

## 2. Methods

### 2.1. Search Strategy

Studies published on the Pubmed, Embase, Cochrane Library, Web of science, Chinese National Knowledge Infrastructure (CNKI), VIP, Wanfang, China Biomedical Literature Database (CBM), and Chinese Clinical Trial Registry System were searched since their time of establishment to September 2018. The search keywords used were “Premenstrual syndrome”, “Premenstrual Tensions”, “Premenstrual”, “Acupuncture”, “Acupoints”, as well as “random” and there was no restriction on language. More details have been provided on the “PROSPERO” website, and the registration number is CRD42018109724.

### 2.2. Study Selection

Types of study: The studies included were RCTs which is stated by the “randomization” phrase, and blinding was not restricted. Case reports, animal mechanism studies, self pre- and postcontrol studies, or non-RCTs were excluded.

Types of participants: Patients with clinically diagnosed premenstrual syndrome were included. Patients who experienced single symptoms such as dysmenorrhea before menstruation were excluded. There was no limitation on age or nation. Patients with severe medical conditions were excluded.

Types of interventions: RCT using acupuncture, acupuncture catgut embedding or acupoint injection with drugs, placebo acupuncture, or no treatment (waiting-listed) for comparison. This review is restricted to acupuncture manipulation, emphasis on penetrating into skin, and manipulating to deqi. Trial on auricular point, acupressure, and laser acupuncture were excluded.

Types of outcome measures: The primary outcomes were effective rate after treatment, effective rate after one month of follow-up, and effective rate of acupuncture treatment with different intervention time. Most of the included studies calculated the effective rate using the reduced symptom scores. The secondary outcome is frequency of acupoints used in the prescription and adverse events including fainting, broken needle, hematoma, and pneumothorax.

Articles that were not available after contacting their authors were excluded. For articles that were repeatedly published, the one with the highest quality was chosen. Articles published for different indicators in the same study were merged.

### 2.3. Data Extraction (Selection and Coding)

Literature screening, study selection, and data extraction were performed by two reviewers (Jiayuan Zhang, Liu Cao). The data extraction was conducted in a standard form, which included characteristics of the study type, patient, intervention details, outcomes, and adverse events. Disagreements were solved by discussion until a consensus was reached.

### 2.4. Data Analysis

Data analysis was performed using Review Manager 5. 3 software provided by the Cochrane Collaboration. Effective rate was calculated by odds risk while heterogeneity was considered to be significant when I^2^ ≥ 50%. A fixed-effect model was performed when there was no significant heterogeneity; otherwise, a random-effect model was performed. Public bias was estimated by symmetry of the funnel plot when the number of the included studies was more than 10.

## 3. Result

### 3.1. The Results of Literature Search and Screening

**Figure 1:**
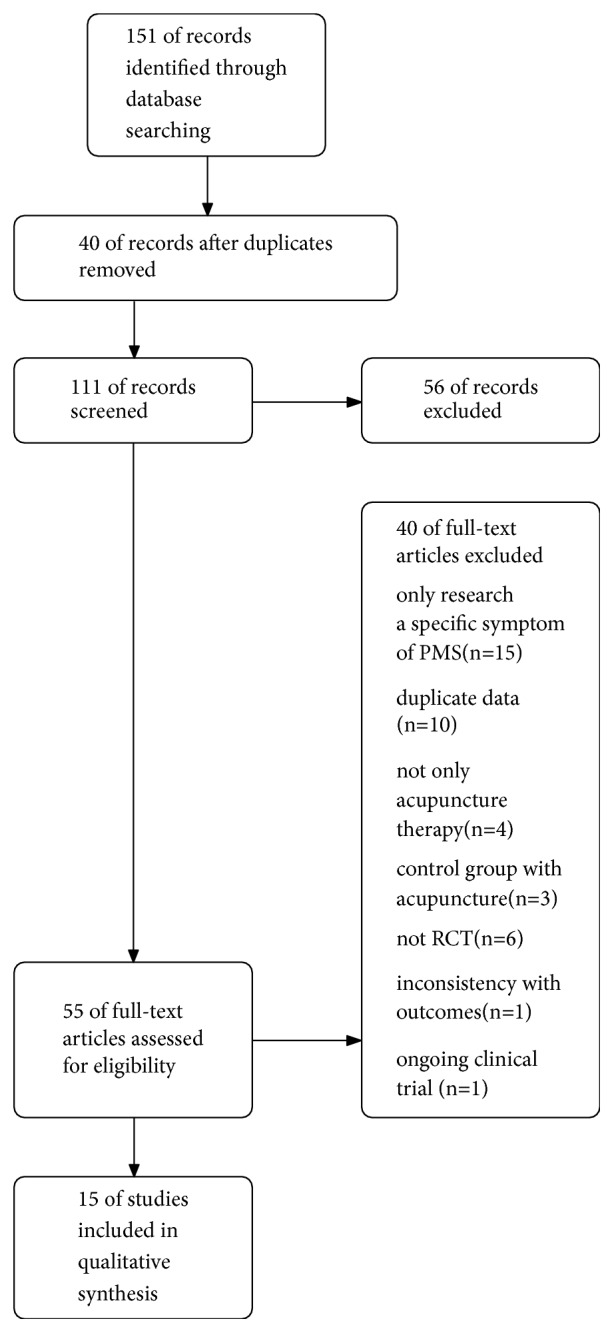
Flow of literature screening.

After searching 9 databases, 151 related articles were collected, and 40 duplicate articles were excluded. A total of 56 articles were excluded because they did not meet the inclusion criteria after reading the title and abstract. The full text of the remaining papers was obtained from the databases, and 40 articles were excluded after full reading and the details are presented in Figure 1. According to the inclusion criteria, 15 articles were finally included, among which 12 were Chinese articles [10–21], 2 were English articles [22, 23], and 1 was Korean literature [24].

### 3.2. Study Characteristics

Twelve studies were conducted in China [10–21], two were conducted in Korea [23, 24], one was conducted in Croatia [22], and all the studies involved a total of 1103 patients with PMS. Various acupuncture techniques were adopted: one study used acupoint embedding [10], one used acupoint injection [11], one used Korean hand acupuncture [23], and one used electroacupuncture [16], while the remaining 11 studies used ordinary acupuncture. The degree of disease was mainly moderate to severe. All the RCTs reported the therapeutic interventional time, in which 6 trials [10, 12, 15, 17, 18, 22] chose 2 weeks (including 14-16 days) before menstruation to start the treatment, 2 trials [11, 21] chose 10 days before menstruation, 2 trials [13, 14] chose 7 days before menstruation, and 1 study [19] reported the treatment was started as the symptoms appeared, while 4 trials [16, 20, 23, 24] treated PMS at a fixed frequency, and the details are summarized in Table 1.

### 3.3. Risk of Bias in Included Studies

**Figure 2:**
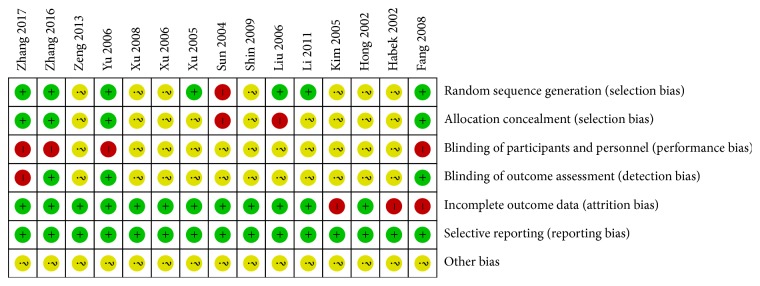
Risks of bias assessment.

As shown in Figure 2, 7 [10–15, 20, 25] studies reported random assignment methods, while 4 trials [12, 14, 15, 20] reported allocation concealment with envelope. Due to the application nature of acupuncture treatment, patients and acupuncturists could not be blinded. The blinding of data collection, analysis, and outcome assessment was reported in 3 studies [12, 14, 20]. Three articles [12, 22, 24] reported unbalanced proportion of dropout in acupuncture group and control group, because the reasons were not given or clearly described as this could affect the final findings. Selective reporting was not found in the included studies.

### 3.4. Outcomes

#### 3.4.1. Acupuncture versus Medication (*n* = 10)

**Figure 3:**
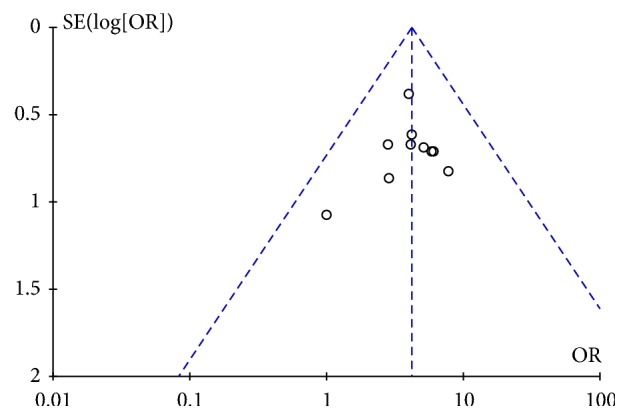
Funnel plot of AT vs medicine.

**Figure 4:**
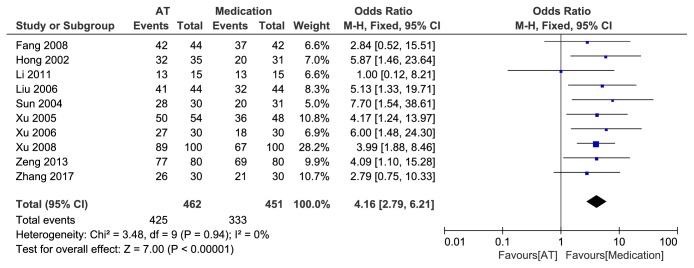
Forest plot of AT vs medicine.

Ten studies [10–13, 15–19, 21] reported the effective rate in acupuncture group compared with medication group. There was no heterogeneity among the studies (P = 0.94, I^2^ = 0%). No obvious funnel plot asymmetry was found (Figure 3) and, thus, no evidence of publication bias was detected. When a fixed-effect model was employed, the pooled results showed a significantly high effective rate due to acupuncture as compared with medication (OR = 4.16, 95% CI (2.79, 6.21), P<0.00001) (Figure 4).

#### 3.4.2. Acupuncture versus Sham Acupuncture (*n* = 3)

**Figure 5:**
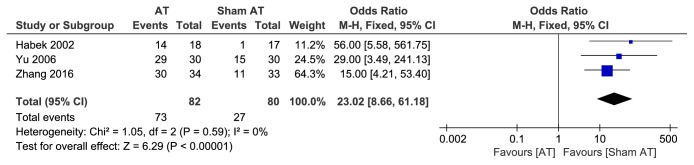
Forest plot of AT vs sham AT.

Three studies [14, 20, 22] reported the effective rate in the acupuncture group compared with sham acupuncture. The pooled results showed a significantly higher efficacy (OR = 23.02, 95% CI (8.66, 61.18), P<0.00001) in acupuncture treatment compared with sham acupuncture with no heterogeneity (P = 0.59, I^2^ = 0%) (Figure 5).

#### 3.4.3. Effective Rate after One Month of Follow-Up (*n* = 2)

**Figure 6:**
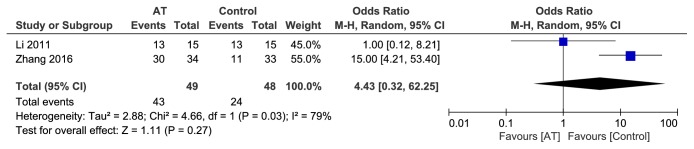
Forest plot of AT vs control after 1 month of follow-up.

Effective rate after follow-up for one month was reported in 2 trails [20, 26]. Since the heterogeneity among the studies was high (P = 0.03, I^2^ = 79%), a random effect model was employed. The pooled results showed that acupuncture did not significantly improve the effective rate compared with control group after 1 month of follow-up (OR = 4.43, 95% CI (0.32, 62.25), P = 0.27) (Figure 6).

#### 3.4.4. Subgroup Analysis of Intervention Time (*n* = 13)

**Figure 7:**
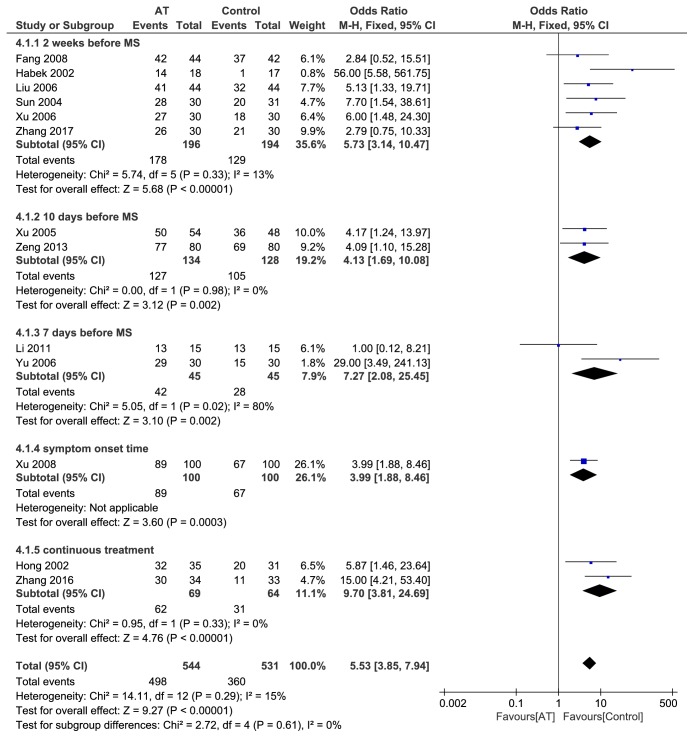
Forest plot of subgroup analysis of different intervention time.

To analyze the difference in clinical curative effect of acupuncture on PMS at different intervention times, subgroup analysis was conducted for the outcome of effective rate after acupuncture therapy at two weeks, 10 days, 7 days before menstruation, or at the symptom onset time. Even with continuous therapy at a fixed-frequency, there was no significant difference between acupuncture group and control group in terms of improving the effective rate for PMS (P = 0.61) (Figure 7).

#### 3.4.5. Frequency of Acupoints (*n* = 13)

Shin [23] used Korea hand acupuncture and Hong [16] used scalp acupuncture; thus, there was no traditional Chinese acupoints in 2 trails. The remaining trails used 37 acupoints, and the frequency was summarized as follows: SP6 (10), LR(7), RN4 (6), RN6, DU20 (5), LI4 (4), PC6, BL18, RN17 (3), EX-HN5, GB20, RN12, RN10, EX-HN3, BL23 (2), ST36, DU24, SP15, ST29, SP10, HT7, LR14, DU8, DU6, DU4, BL18, BL47, BL49, BL51, DU15, EX-CA1, KI6, BL15, KI3, RN3, GB34, RN9 (1).

#### 3.4.6. Adverse Events

Three trails reported mild adverse events that did not require treatment. Yu [14] reported that 2 patients had hypomenorrhea at the second menstruation cycle but recovered at the third cycle in acupuncture group. Zhang [20] reported 7 cases of adverse events including pain and hematoma among 3 patients in acupuncture group, pain among 2 patients in nonacupoint superficial group, and itching and flush of the skin and pain among 2 patients in sham acupuncture group. Habek [22] reported that 1 patient had haematoma in acupuncture group. Shin [23] reported that no adverse events occurred. The remaining trials did not mention any adverse events.

## 4. Discussion

### 4.1. Analysis of Efficacy

This systematic review shows that the overall effectiveness of acupuncture treatment of premenstrual syndrome is superior to that of sham acupuncture and related medications, which is consistent with previous systematic review reports [5, 6]. Modern neurophysiological studies have shown that acupuncture may promote the release of specific neuropeptides by stimulating the central nervous system, causing important physiological effects, even activating self-repair mechanisms [27]. Acupuncture manipulation or electrical stimulation of fine needles pierced into the skin and muscle activates A*α*, *β*, *δ*, and cholinergic fibers [28], thereby regulating autonomic nervous system activity [29]. Traditional Chinese medicine believes that “the balance of Yin and Yang” is the key to being health. Acupuncture regulates blood circulation and balances the yin and yang through local stimulation of acupoints.

As early as 2006, the complex and close interactions between metabolism and immunity were considered to be key mechanisms for the body to maintain homeostasis [30]. Many disease processes are accompanied by abnormalities in metabolism and immunity. Therefore, “Nature Reviews Immunology” officially proposed the concept of immunometabolism [31], which has become a hot spot in the scientific community. The theory of traditional Chinese medicine (TCM) “yin -yang balance theory” has certain similarities. TCM theory believes that the nutrient metabolism in modern medicine belongs to Yin while the immunoinflammatory reaction belongs to “Yang.” The balance of interaction between immunity and metabolism represents the balance of “yin and yang, qi, and blood” to a certain extent, while the acupuncture treatment of traditional Chinese medicine achieves the balance of yin and yang by stimulating local acupoints.

### 4.2. Analysis of Intervention Time

The intervention time of acupuncture treatment for PMS in the included studies varied across intermenstrual, premenstrual, and entire menstrual cycles. 6 of the 15 clinical studies began treatment of PMS about 2 weeks before menstruation, 2 were 10 days before menstruation, 2 were 7 days before menstruation, while 1 started acupuncture treatment when the symptoms of PMS appeared. Four of the 15 clinical studies employed continuous treatment for premenstrual syndrome. It was found that, after acupuncture treatment at different times before the menstrual treatment, the difference in efficacy was not statistically significant based on subgroup analysis. An authoritative standard for the intervention time of Chinese medicine treatment for PMS has not been setup.

It has been reported that the brain responded differently to the immediate and sustained stimulation of acupuncture [32, 33], which infers that the timing of acupuncture treatment has important influence on the efficacy. Several researchers have reported the intervention time in treating menstrual cycle-related diseases, but the results of these clinical studies vary widely [25, 34–36]. The difference in the efficacy of early acupuncture and immediate acupuncture is controversial. Animal studies have shown that immediate acupuncture has a significant immediate analgesic effect [26], while early acupuncture has a more pronounced role in regulating the hypothalamic-pituitary-gonadal state [37]. In general, immediate acupuncture has a strong effect in improving symptoms immediately, while early acupuncture is good at regulating the functional state of the body. According to the concept of modern time medicine, “people are corresponding to the nature,” the laws of the human body change with nature, and the therapeutic effect of acupuncture points can be enhanced by selecting the best time for acupuncture intervention therapy, and finally stimulating the self-adjustment function of the body [38]. A subgroup analysis of intervention time of acupuncture was thus conducted. However, according to this systematic review, there were no significant differences among different intervention times for starting acupuncture treatment for PMS. Maybe there is no need for restriction on the choice of time points; the intervention time can be selected according to the specific state of the patient. Immediate acupuncture is used to improve symptoms, and early acupuncture is used to adjust the overall state, which is also in line with the treatment principle of Chinese medicine: “treat the manifestation for emergency cases, treat root in chronic cases.”

Furthermore, considering the small sample size, short follow-up duration, and little observation time points of the included trials, the cumulative effect of early acupuncture on PMS may not be apparent. Consequently, studies on the timing of acupuncture intervention in patients with PMS are indispensable, but the sample size should be expanded, the follow-up time should be prolonged, and the time point of efficacy observation should be increased.

### 4.3. Frequency Analysis of Acupoints in Treating PMS

SP6 was the most frequently used acupuncture point in this review. TCM theory states that SP6 is the intersection of the three yin meridians of the liver, the spleen, and the kidney. The occurrence of gynecological diseases is closely related to these three internal organs; SP6 is thus the key point for the treatment of gynecological diseases. Presently, studies on acupuncture at SP6 for PMS mainly focus on the nervous system. Modern research shows that the incidence of PMS may be related to abnormal neural activity in the brain's default mode network (DMN) [39, 40]; thus, acupuncture treatment at SP6 can regulate abnormal nerve activity in the DMN area [41]. In addition, animal studies have shown that acupuncture at SP6 and RN4 can regulate the hormone balance of the hypothalamic-pituitary-ovarian axis [42]. The other two acupoints with higher frequency are LR3 and Ren4. According to the results of this review, the most used acupuncture points in treating PMS are SP6, LR3, and RN4.

### 4.4. Security Analysis

Three of the 15 studies reported adverse reactions of acupuncture (hypomenorrhea, subcutaneous hematoma, and pain), while the other 12 studies did not mention the adverse reactions. Therefore, the safety of acupuncture could not be judged comprehensively in this review. However, it can preliminarily be inferred that acupuncture is safe to some extent according to the results of this study.

### 4.5. Limitations of Study

The methodological quality of the literature included in this study is poor, and the sample size of each study is small. There is no standard guideline for the acupuncture treatment and the evaluation system of its efficacy. Due to the particularity of TCM- (traditional Chinese medicine-) related research, most of the literatures included in this review were conducted in China, which presents a possible language bias. Therefore, the findings of this study should be interpreted cautiously. Large-scale, case-control studies with rigorous designs are necessary to provide accurate evidence.

## 5. Conclusion

In conclusion, acupuncture treatment leads to higher effective rate for PMS. There is no significant difference of effective rate in intervention time of acupuncture treating PMS. The most used acupoints for PMS treatment are SP6, LR3, and RN4. However, considering the methodological limitation of the studies, caution needs to be taken in applying the conclusions of this review. Thus, further studies with high methodological quality are required to validate the conclusions of this review.

## Figures and Tables

**Table 1:** Characteristics of the included studies.

Study	Sample E/C	Experiment	control	Acupoints	Intervention time	Outcomes	AE
Liu 2006 [10]	44/44	ACE	Medicine	PC6, SP6, CV4, CV17, LR3	15 days before MS	IR	NR

Xu 2005 [11]	54/48	AI+AT	Medicine	AI in SP6, ST36 and AT in L14, LR6, RN4, RN6, EX-HN5	10 days before MS	IR	NR

Fang 2008 [12]	47/43	AT	Medicine	DU24, LR3+L14, SP6	14-16 days before MS	IR, symptom score, HAMA score	NR

Li 2011 [13]	15/15	AT	Medicine	RN12, RN10, RN4, RN6, SP15, ST29	7 days before MS	symptom score, SCL-90 scores, IR	NR

Yu 2006 [14]	33/32	AT	sham AT	DU20, EX-HN3, EX-HN5, SP6, SP10	7 days before MS	symptom score Uncomfortable Days IR	hypomenorrhea

Zhang 2017 [15]	30/30/30	AT	Medicine/ACE+Auricular points	LR3, SP6, PC6, LA14, BL18	14 days before MS	symptom score SCL-90 score IR, E2, P	NR

Hong 2002 [16]	35/31	EA	Medicine	Scalp acupuncture: Frontal line, middle line of vertex	three times per week	symptom score, IR	NR

Xu 2006 [17]	30/30	AT	Medicine	DU8, DU6, DU4, BL18, BL20, BL22, BL47, BL49, BL51	14 days before MS	IR	NR

Sun 2004 [18]	30/31	AT	Medicine	D20, RN17, RN4, SP6, PC6, LR3	14 days before MS	symptom score, IR	NR

Xu 2008 [19]	100/100	AT	Medicine	GB20, DU15	When the symptoms occurred	IR	NR

Zhang 2016 [20]	35/35/35	AT+Intradermal needle	non acupoint superficial AT sham AT	AT:DU8, EX-HN3, RN12, RN10, BL24, RN4, EX-CA1, SP6, KI6, LI4, LR3 Intradermal needle:BL15, BL18, BL23	AT:3 per week Intradermal needle:2 times per week	HAMA/WHOQOL-BREF/DRSP scale IR	Hematoma, pain;redness, itching, in the skin, pain

Zeng 2013 [21]	80/80	AT+Medicine	Medicine	LR3, K13, RN6, BL18, RN17, SP6	10 days before MS	IR	NR

Habek 2002 [22]	18/17	AT	sham AT	DU20, L14, LR3, RN3, RN4, RN6, PC6, GB34, BL23	Luteal phase	IR	Hematoma

Shin 2009 [23]	10/10/10	Korea hand AT	no treatment	A5, A6, A8, A12, A16, A18, N18, F6	1 per 3 days	symptom score	None

Kim 2005 [24]	10/10	AT	sham AT	SP6, RN9	2 per week	symptom score	NR

(ACE: acupuncture catgut embedding, A/C: acupuncture group versus control group, AE: adverse events, AI: acupoint injection, AT: acupuncture, DRSP score: Daily record of severity of problems scale, E2: estradiol, ET: electroacupuncture, HAMA: Hamilton anxiety scale, IR: effective rate, MS: menstruation, NR: not reported, P: progesterone, SCL-90: SymptomChecklist90, WHOQOL-BREF scale:WHO Quality of Life-BREF scale)
